# Artifact filtering application to increase online parity in a communication BCI: progress toward use in daily-life

**DOI:** 10.3389/fnhum.2025.1551214

**Published:** 2025-03-04

**Authors:** Tab Memmott, Daniel Klee, Niklas Smedemark-Margulies, Barry Oken

**Affiliations:** ^1^Department of Neurology, Oregon Health & Science University, Portland, OR, United States; ^2^Institute on Development and Disability, Oregon Health & Science University, Portland, OR, United States; ^3^Khoury College of Computer Sciences, Northeastern University, Boston, MA, United States; ^4^Departments of Behavioral Neuroscience and Biomedical Engineering, Oregon Health & Science University, Portland, OR, United States

**Keywords:** EEG, N200 and P300 potentials, artifact handling, signal processing, cBCI, brain computer interface, online parity, signal filtering

## Abstract

A significant challenge in developing reliable Brain-Computer Interfaces (BCIs) is the presence of artifacts in the acquired brain signals. These artifacts may lead to erroneous interpretations, poor fitting of models, and subsequent reduced online performance. Furthermore, BCIs in a home or hospital setting are more susceptible to environmental noise. Artifact handling procedures aim to reduce signal interference by filtering, reconstructing, and/or eliminating unwanted signal contaminants. While straightforward conceptually and largely undisputed as essential, suitable artifact handling application in BCI systems remains unsettled and may reduce performance in some cases. A potential confound that remains unexplored in the majority of BCI studies using these procedures is the lack of parity with online usage (e.g., online parity). This manuscript compares classification performance between frequently used offline digital filtering, using the whole dataset, and an online digital filtering approach where the segmented data epochs that would be used during closed-loop control are filtered instead. In a sample of healthy adults (*n* = 30) enrolled in a BCI pilot study to integrate new communication interfaces, there were significant benefits to model performance when filtering with online parity. While online simulations indicated similar performance across conditions in this study, there appears to be no drawback to the approach with greater online parity.

## Introduction

### Background

Brain-Computer Interfaces (BCIs) have emerged as a promising technology for facilitating communication and control between the human brain and external devices (Kübler, 2020; Wolpaw and Wolpaw, 2012). BCIs hold immense potential to enhance quality of life for individuals with diverse needs and may allow individuals to perform a wide range of tasks, including typing messages, controlling a wheelchair or robotic arm, selecting items from a menu, or playing video games (Allison et al., 2020; Kawala-Sterniuk et al., 2021).

In particular, BCI systems can potentially improve the quality of life for people with severe disabilities by restoring their ability to communicate and interact with the world around them. BCIs for communication (cBCIs) enable individuals with severe motor disabilities, such as quadriplegia or locked-in syndrome, to communicate with the outside world using their brain signals. cBCIs detect and interpret specific patterns in the user’s brain activity, which are then translated into commands that can control a computer or other electronic device to generate speech, text, or other forms of communication output, and which can be customized to the user’s specific needs and preferences. Like other Augmentative and Alternative Communication (AAC) devices, cBCIs can provide individuals with a means of expressing themselves and engaging in social interactions, which can have a significant positive impact on their quality of life (Kübler et al., 2009; Milekovic et al., 2018; Peters et al., 2022; Pitt et al., 2022; Pitt and Brumberg, 2023). These systems may be implantable, requiring surgical intervention with the benefit of higher signal quality, or non-implantable, low-risk sensors external to the end-user yet subject to higher electrical or other interference. While the implantable BCIs are approaching a speed closer to spoken text and are a promising technology, the need for non-surgical and low-cost options will remain an area of interest to both end-users and researchers (Willett et al., 2021).

Several brain signals and interfaces can be leveraged for non-implantable cBCIs (Peters et al., 2022; Akcakaya et al., 2014). Many rely on event-related potentials (ERPs), like the N200/P300 attentional responses. These mechanisms are often paired with typing interfaces such as the Matrix Speller or Rapid Serial Visual Presentation (RSVP) (Akcakaya et al., 2014; Acqualagna and Blankertz, 2013) to facilitate communication. However, the successful implementation of cBCI systems faces significant challenges, primarily due to the presence of artifacts in the acquired brain signals. Artifacts, which are unwanted signals or noise, can degrade the accuracy and reliability of BCIs (Awais et al., 2024; Jafarifarmand and Badamchizadeh, 2019; McDermott et al., 2022; Minguillon et al., 2017).

Artifact handling in BCI systems generally involves a series of steps: avoidance, detection, removal, and, if necessary, the reconstruction of the EEG data. Artifact avoidance can include experimental design modifications (e.g., using shielded rooms or adjusting the distance from electromagnetic sources), user instructions (e.g., minimizing blinks), or participant screening procedures. While avoiding artifacts appears to be the most straightforward approach, such methods may not be practical for real-world BCI applications, which are inherently noisier than controlled laboratory environments. Furthermore, even common instructions—such as asking users to refrain from blinking—can lead to reduced control signal amplitudes or increased mental fatigue (Magliacano et al., 2020; Ochoa and Polich, 2000; Oken et al., 2018). Artifact filtering, which involves removing or attenuating non-brain signal frequencies, is another common approach. However, the choice of filter—particularly the frequency cutoff and filter order—can impact performance. If not applied correctly, filtering can either introduce unwanted noise or inadvertently remove important brain activity. While most filtering approaches fall within the 0.1–75 Hz range, there is as-yet no consensus on the best filter type, and no “one-size-fits-all” solution has emerged in the literature (Zhang et al., 2024; Zhang et al., 2024a). It may be fruitful then to distinguish between artifact filtering applied offline (after data collection) and online hardware filtering, which is done during EEG acquisition to avoid losing information due to the Nyquist limit for sampling frequency, or due to amplifier saturation as a result of very low frequency activity shifts in baseline. Furthermore, studies have shown that removing or reconstructing data contaminated with artifacts can have the opposite of the intended effect and potentially lower performance (McDermott et al., 2022; Delorme, 2023; Thompson et al., 2019), while others show benefits (Zhang et al., 2024b). In any case, the impact of these artifacts on BCI has proven detrimental (Awais et al., 2024).

### Review of relevant literature

Despite extensive research on artifact handling in BCIs (McDermott et al., 2022; Minguillon et al., 2017; Guarnieri et al., 2018; Kim and Kim, 2018; Liu et al., 2021; McDermott et al., 2023; McFarland et al., 2005; Vaughan et al., 2006), and in cognitive neuroscience more generally (Zhang et al., 2024a; Delorme, 2023; Zhang et al., 2024b; Islam et al., 2016; Jiang et al., 2019; Saba-Sadiya et al., 2021; Urigüen and Garcia-Zapirain, 2015), a clear best practice has yet to emerge for existing interfaces. That is, researchers generally acknowledge the problematic nature of EEG artifacts and their potential to detrimentally affect control signals, but there has been no consensus on how or when to implement corrective actions in BCIs. Handling artifacts for BCIs may be especially challenging, since these steps must necessarily be performed in a real-time, closed-loop fashion, and the artifact handling approach must be resilient to sample loss, time constraints, and potential processing resource limitations. A crucial yet often overlooked aspect of BCI design is “online parity”—the need for processing conditions to match those applied during real-time use. This principle was advocated for in terms of practicality for use in daily-life by Minguillon et al. (2017), who argued that, for a BCI to be practically useful in daily life, it must be able to operate online with acceptable delays. While many studies adopt artifact handling procedures from co neuroscience (often filtering data offline), these approaches have not been systematically evaluated for their effectiveness in closed-loop BCI systems. It may be tempting to pull best practices from the cognitive neuroscience literature, particularly in a calibration task that can be trained offline with filtering applied to the whole session (referred to as “conventional” filtering in this manuscript). For optimal performance and transferability, signal models would ideally be trained on the same data, processed in the same way, and under the same conditions as during online use. A review of all literature used in this manuscript reveals that the majority of relevant studies rely on this conventional filtering approach (Kübler et al., 2009; Akcakaya et al., 2014; Acqualagna and Blankertz, 2013; Awais et al., 2024; McDermott et al., 2022; Oken et al., 2018; Thompson et al., 2019; McDermott et al., 2023; Hoffmann et al., 2008; Mowla et al., 2020).

Other studies have employed adaptive filtering or online calibration techniques, which effectively ensure adherence to the online parity principle (Guarnieri et al., 2018). Furthermore, more advanced methods of artifact reconstruction may be used to replace missing or corrupted data. Techniques include independent components analysis, principal components analysis, empirical mode decomposition, and canonical correlation analysis. These techniques have been shown to improve accuracy in some settings, such as motor imagery classification (Ferracuti et al., 2022), while others have demonstrated detrimental effects, such as in P300 applications (Thompson et al., 2019). These techniques, while promising, are currently limited in their online applicability; they may require high processing resources, computation time, or manual intervention to select the right components. Additionally, the transferability of these components between sessions has not been thoroughly evaluated and could be limiting given the lack of stationarity of the underlying EEG signals.

Several modeling techniques have become standard of practice, and research continues to make incremental progress in the classification of P300 and other signals for use in BCIs (Mowla et al., 2020). When implementing artifact handling procedures, it is important to consider the role of signal modeling. For instance, signal models with decomposition or processing components, such as neural network layers or component analyses, can act as processors for the underlying signals and may function well without pre-processing (Alzahab et al., 2021). Alzahab et al. (2021) in their systematic review of deep learning BCIs reported 21% of papers using the technique did no preprocessing whatsoever while maintaining accuracy. Furthermore, these approaches have had limited success in transferring to group or population models, likely due to the high signal and cognitive variability between users and sessions (Höller et al., 2013). Considering the unknown underlying information used for classification and the impact of decomposition layers, the effect of artifact handling procedures on different models remains unclear.

In summary, the literature pertaining to artifact handling in BCIs has not reached consensus on best practice. Furthermore, the use of neural or decomposition components in a signal modeling pipeline may add further confounds to this goal. The majority of studies reviewed pre-process data in a conventional way without justification for the practice or analysis to show whether online parity processing would be contraindicated.

### Hypothesis

The present study aims to advance the literature on artifact handling in BCIs by investigating the concept of online parity in signal filtering. We hypothesize that a filtering approach that aligns with the conditions of online use will lead to better classifier accuracy compared to an approach that mismatches online and offline filtering. Specifically, we compare the conventional filtering approach (CF) to an online filtering approach (OF), examining their effects on classifier accuracy across several established BCI signal models.

## Methods

We used P300 spelling calibration data from a convenience sample pilot study involving 31 participants without disabilities (mean age = 49 ± 20 years) (Peters et al., 2025). One participant was excluded due to hardware failure. Data were collected in an office environment at Oregon Health & Science University (OHSU). The pilot study consisted of a single visit, during which participants completed an RSVP calibration task, followed by RSVP copy phrase tasks (more details below). Some of the original copy phrase tasks included switch inputs to test a new typing interface. For the primary analysis, we used the calibration data from 30 participants. In subsequent simulations of copy-phrase data, data from six participants were not available due to low calibration accuracy (< 0.70 AUC) or hardware failure in the original study (*n* = 25) (Table 1).

**Table 1:** Participant demographics.

	*n* = 30
Age (years)	
Mean ± SD (range)	48.83 ± 20.06 (19–82)
Gender	
Female	18
Male	12
Race	
American Indian or Alaska Native	2
Black or African American	1
Asian or Asian American	3
Caucasian	22
Other/Multiple	2
Ethnicity	
Hispanic/Latino	2
Not Hispanic/Latino	28
Education	
High School/GED	1
Some college	4
Associate degree	2
Bachelor’s degree	8
Postgraduate degree	15

### Task

**Figure 1:**
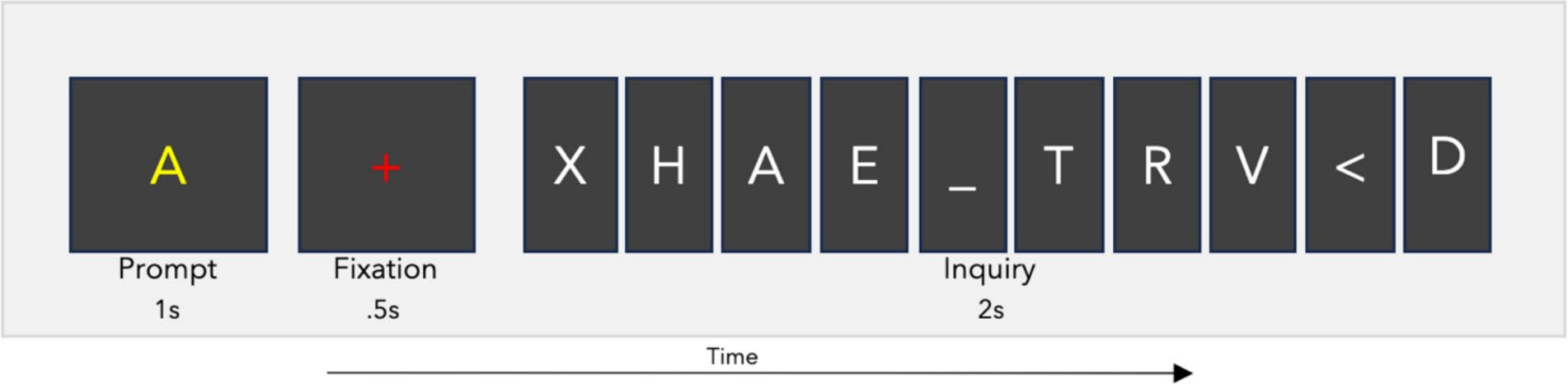
RSVP calibration task. The RSVP calibration task prompts a user to search for a letter in an inquiry. This one-second prompt is followed by a fixation of 0.5 s, and then the 10-character inquiry. Each inquiry was presented at a rate of 5 Hz for a total time of two seconds per inquiry. The full iteration lasts 3.5 s. The user completed this process 110 times with a four second blank screen between inquiry iterations.

The RSVP calibration task presented letter characters at a rate of 5 Hz, with 110 inquiries consisting of 10 letters each (for a total of 1,100 trials) using BciPy (Memmott et al., 2021). The stimuli included all 26 letters of the English alphabet, as well as the characters “_” for space and “<“for backspace. In 10% of the inquiries, only non-target characters were shown. The sequential order of target stimuli was randomly distributed among the 10 possible positions across the 110 RSVP inquiries. Between inquiries, there was a two-second blank screen. Each inquiry consisted of a one-second prompt showing the target letter, followed by a 0.5-s fixation, and then the presentation of the 10-letter inquiry (see Figure 1). The letters were displayed in the center of the screen using the Overpass-Mono font, in white on a black background. Target prompts were yellow, while fixation crosses were rendered in red.

After completing the RSVP calibration task, participants performed several rounds of a copy spelling task using different user interfaces (RSVP copy phrase task). In this online task, participants were instructed to spell a predetermined five-letter word within a larger phrase. For example, in the phrase “I want to go to the store,” they would be asked to type “store” using the BCI system. Participants could select the backspace character (“<“) if they made an incorrect selection. The experimental copy phrase included three Inquiry Preview (IP) conditions and one No Preview condition. In the IP conditions, a box with symbols appeared before the fixation to alert the participant to the upcoming inquiry. The two of the three IP conditions allowed participants to confirm or skip the upcoming inquiry using a button press. For the present analysis, only the No Preview condition of the copy phrase task was used. Each participant completed four copy phrases in this condition, and the order of conditions was randomized for each participant.

### Data acquisition

EEG data were collected using the DSI-24, dry electrode cap (Wearable Sensing, San Diego CA) at a sampling rate of 300 Hz. The device employs a hardware filter permitting a collection bandwidth of 0.003–150 Hz. Data were recorded from Fp1/2, Fz, F3/4, F7/8, Cz, C3/4, T7/T8, T3/T4, Pz, P3/P4, P7/P8, T5/T6, O1/2 with linked-ear reference (A1 and A2) and ground at A1. All data were collected using a Lenovo Legion 5 Pro Laptop with Windows 11, an Intel Core i7-11800H @ 2.30 GHz, 16 GB DDR4 RAM, and a NVIDIA GeForce RTX 3050. Trigger fidelity on the experiment laptop was verified using the RSVP Time Test Task in BciPy and a photodiode. The results of this timing test were used to determine static offsets between hardware and prevent experimentation with any timing violations greater than +/− 10 ms. All software was written by the research team and is freely available on GitHub or PyPi using BciPy version 2.0.1rc4 (pip install bcipy==2.0.1rc4).

### Filters

**Figure 2:**
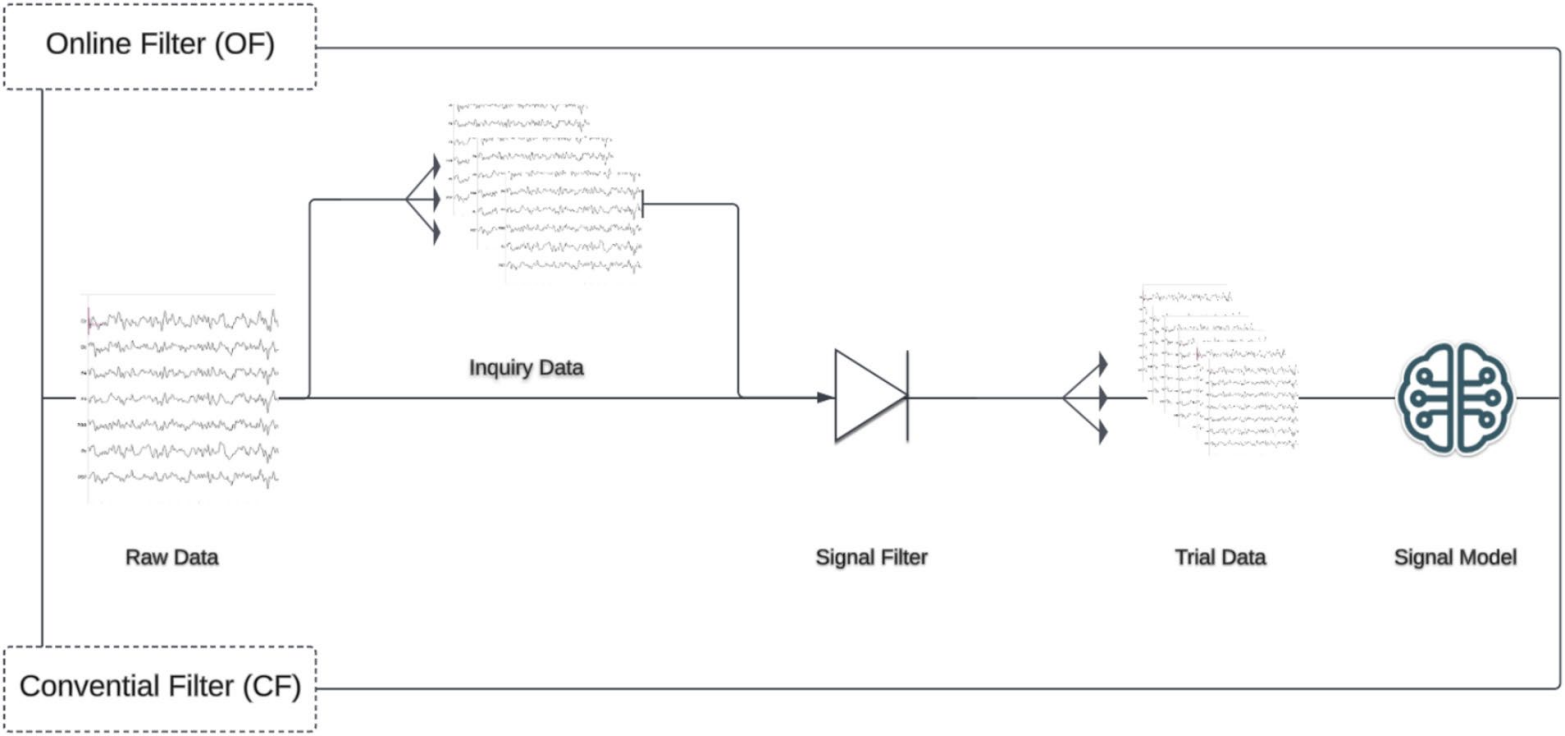
Online and conventional filter application. This study examines two filtering pipelines, each with several similar steps. However, the OF condition requires an additional epoching step before filtering. The top of figure illustrates this distinction. In the OF condition, raw data is first epoched into Inquiry Data before filtering, then further epoched into Trials, and finally passed into a Signal Model. In contrast, the CF condition filters the data immediately, epochs it into trials, and then passes it to a Signal Model for training.

To investigate the impact of filtering procedure on model performance, two filtering pipelines were constructed. The first, which was labeled as conventional filter (CF), applies a signal filter to the whole calibration dataset and then segments the data into target/non-target trials. This approach represents the current state of EEG processing across disciplines. Next, an online filter (OF) was constructed in which the calibration datasets were reshaped into inquiries with a one second buffer on each end, filtered, and then divided into trials (target, non-target) for classification. The signal filters consisted of the following in order: a zero-phase (two-directional) 60 Hz notch filter with quality factor 30, 1-20 Hz bandpass filter, and down sampling by a factor of two. The bandpass filter used was a causal (forward-only) Butterworth IIR filter, 5th order, constructed using Scipy version 1.5.2 (Virtanen et al., 2020). This filter was the default used in BciPy for P300 spellers (Figure 2).

### Signal modeling

The following signal models were used for P300 classification: Linear Discriminant Analysis (LDA), Logistic Regression (LR), and lastly, the default BciPy model, which is a channel-wise Principal component analysis, followed by a Regularized discriminant analysis and Kernel density estimation (PRK). All other models and performance exports besides the default were constructed using scikit-learn (Pedregosa et al., 2011). The models were trained using GridSearchCV over ten-fold cross-validation, exporting the mean of model performance in terms of Balanced Accuracy (BA) and Matthew’s Correlation Coefficient (MCC). The resulting meta parameters for the models were then used for reporting. The LR model used an L2 penalty, L-BFGS solver, and an inverse regularization of 0.0183. The LDA model used Xdawn Covariance and Riemann Tangent Space transformers before inputting into an LDA model (Barachant et al., 2012; Barachant et al., 2013; Coelho Rodrigues et al., 2017). LDA was trained with shrinkage set to auto and solver to eigen. All filtering approaches are trained and tested using the same modeling procedure; for example, when filtering using CF, the model is trained and tested using trials filtered with the same approach.

### Performance metrics

Model performance was measured using BA and MCC. While MCC may be a better measure of performance for these data, both are reported to make comparisons between published studies easier. BA measures the performance of a classifier and supplies a more exact metric than simple area under the curve (AUC) or accuracy, since it considers the unequal class frequencies that are common in BCI interfaces (Brodersen et al., 2010). It is the average of the True Positive Rate (TPR; sensitivity) and True Negative Rate (TNR; specificity).


$$\mathrm{BA}=\frac{TPR+TNR}{2.}$$

MCC was reported to be less affected by large class imbalances when compared to other measures, such as BA (Chicco et al., 2021; Chicco and Jurman, 2020; Matthews, 1975). In situations where one class is rare, MCC provides a more reliable sign of how well a model performs, especially with respect to the minority class. Due to the high ratio of negative to positive class in this study (10:1), this was an ideal measure to avoid type I and II statistical errors. The MCC ranges from −1 to +1, where +1 indicates a perfect prediction, 0 indicates no better than a random prediction, and − 1 indicates total disagreement between prediction and observation. The score can be calculated via the True Positive (TP), True Negative (TN), False Positive (FP), and False Negative (FN) values from the confusion matrix via the following formula:


$$MCC=\frac{\left(TP\times TN\right)-\left(FP\times FN\right)}{\sqrt{\left(TP+FP\right)\left(TP+FN\right)\left(TN+FP\right)\left(TN+FN\right)}}$$

### Simulation

To determine the viability of the different filtering approaches online, a simulation using the PRK models on the copy phrase tasks were run. The copy phrase data were loaded, filtered using OF to simulate the online constraints, and model likelihoods were generated from the saved models in the same way that would be done during a closed-loop experiment in BciPy. These outputs were then scored into a confusion matrix with the following criteria:

*True Positive (TP):* Targets with likelihoods >1.0

*False Negative (FN):* Targets with likelihoods <= 1.0

*False Positive (FP):* Non-targets with likelihoods >1.0

*True Negative (TN):* Non-targets with likelihoods <= 1.0

From these scored values, BA and MCC were calculated using the formula detailed above.

### Statistics

The filter application results were evaluated for significant differences (*p* < 0.05) using a permutations cluster two-tailed *t*-test (50,000 permutations) between model types using MNE-Python (Gramfort et al., 2013). The threshold for permutations were calculated using SciPy percent point function (ppf) with an alpha of 0.05 and 29 degrees of freedom (Virtanen et al., 2020). The resulting Mean, Range and Standard Deviation values are reported alongside *p*-values.

## Results

### Filter application

**Figure 3:**
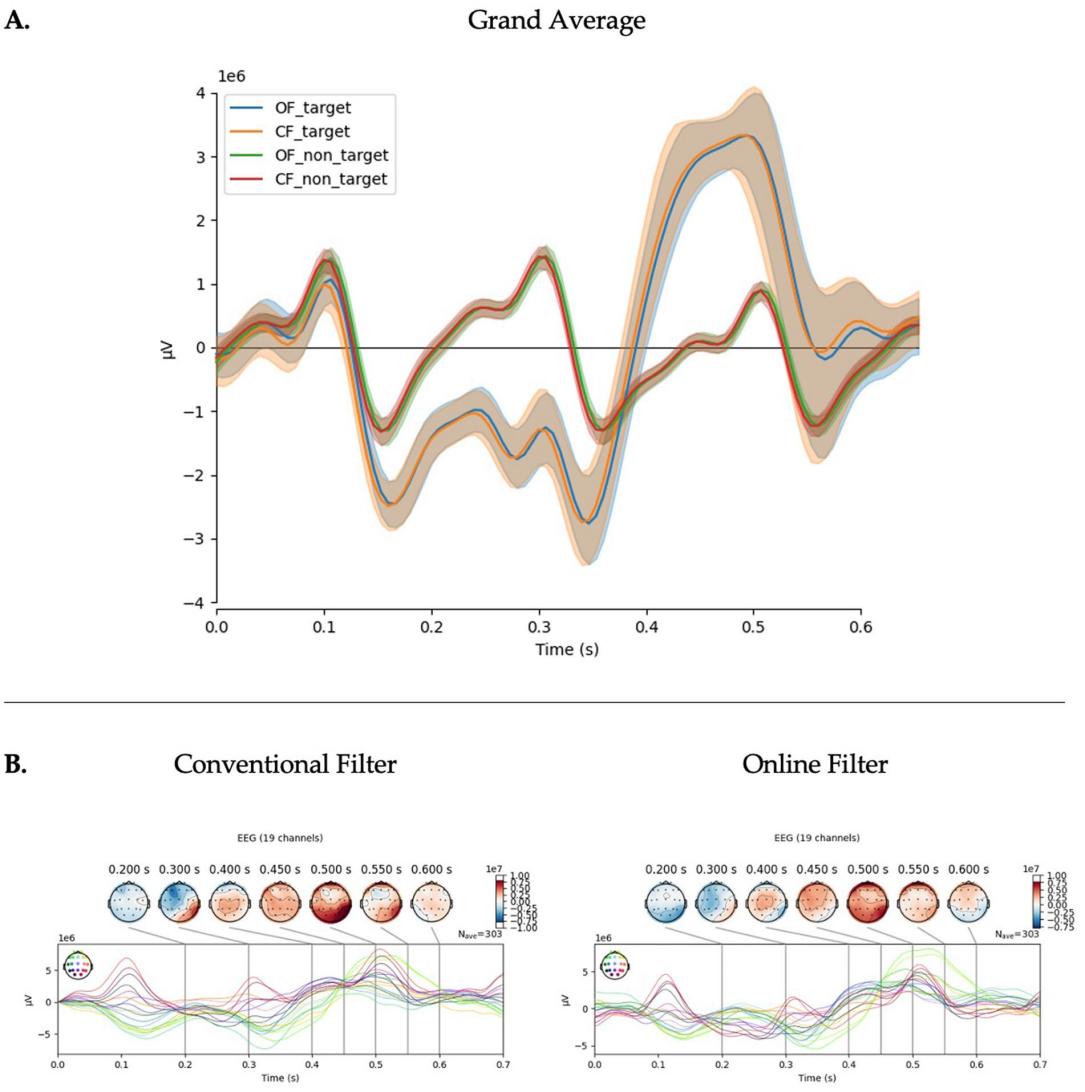
Filter application on EEG signals. **(A)** Grand average of calibration data (*n* = 30) showing the averaged ERPs recorded from channels ‘Pz’, ‘Cz’, ‘Oz’, ‘P3’, ‘P4’, ‘O1’, ‘O2’. This panel shows that the paradigm successfully evoked a P300 response during the target condition that was classifiable for use in online typing. In the target conditions, the orange line represents the conventionally filtered data (CF) and the blue line the online filter (OF). In the non-target conditions, the red line represents CF and the green line OF. There appears no major distinction on group average for the ERP. **(B)** Topographic maps of target condition for CF (left) and OF (right). These demonstrate activity across channels and the impact of the different filtering conditions across channels. The OF produces similar results to CF with some reduction in early potentials (N1, P1) and slight changes in topography.

In the investigation of the filter application procedure, the OF model, which featured greater online parity, yielded the best performance across multiple models and metrics. On average, all model types showed statistically significant improvements with the OF procedure, with the exception of BA in the PRK model, which just trended toward significance (*p* = 0.131). The statistical results are summarized in Table 2 and further examined through group averages (see Figure 3). There were no significant visual differences observed in the ERP plots.

**Table 2:** Filter application model performance.

	LR	PRK	LDA
Matthew’s correlation coefficient			
OF M (SD)	0.229 (0.136)	0.342 (0.175)	0.343 (0.193)
CF M (SD)	0.2163 (0.139)	0.329 (0.172)	0.334 (0.197)
*p*-value	**0.01**	**0.044**	**0.045**
Balanced accuracy			
OF M (SD)	0.638 (0.077)	0.739 (0.11)	0.641 (0.088)
CF M (SD)	0.632 (0.079)	0.733 (0.11)	0.636 (0.088)
p-value	**0.044**	0.131	**0.002**

Classification performance across models and filter application types. Values reported are mean (standard deviation). The OF produced statistically significant improvements across models in the MCC metric. While the trend held using BA, PRK only trended toward significant (*p* = 0.131). All models performed similarly classifying this dataset, however the PRK model performed best across metrics.

Bolded values indicate a significant difference between conditions.

### Supplementary analyses

**Figure 4:**
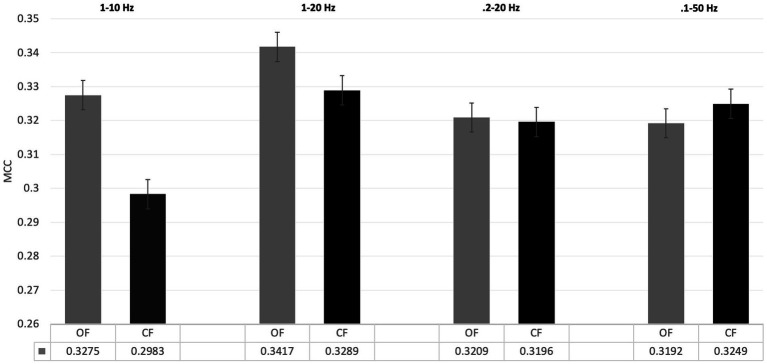
Filter band settings on classification performance. This figure demonstrates the impact of filter band settings on classification performance between filter application conditions. All OF results are shown in grey; CF results are shown in black. Performance was determined with the default PRK model and measured in terms of MCC. Standard error bars applied. Averages are reported at the bottom of each condition. The OF condition provided better or equal performance to the CF condition across all filters. The 1-20 Hz filter performed the best on this dataset across conditions.

**Figure 5:**
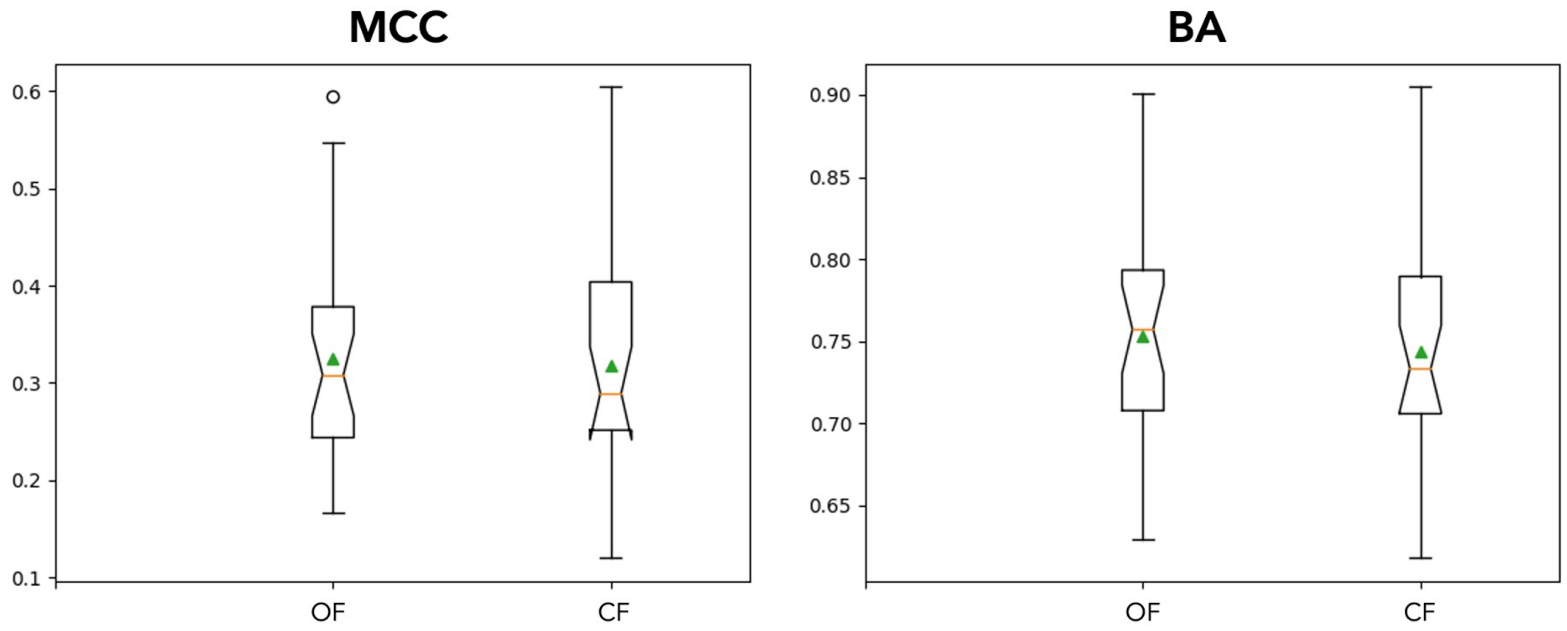
Online simulation results. The above notched box plots demonstrate stimulated online classification performance between filter application conditions across participants. The means are plotted using a green triangle, outliers are denoted with circles above/below the whiskers. In the top plots, MCC (left) and BA (right) are plotted. The models performed similarly with OF having a slight advantage on average.

To complement the aforementioned analyses, two supplementary investigations were conducted: (I) an analysis of varying filter bands, and (II) a simulation using the evaluated models on collected online data from the RSVP copy task.

(I) In the first analysis, different filter bands were applied to the PRK model. Model performance was assessed using MCC (see Figure 4). The following filter bands were tested: 1–10 Hz and 0.2–20 Hz, as recommended by Zhang et al. (2024b) for N200/P300 ERPs; 1–20 Hz, the default filter in BciPy; and 0.1–50 Hz, selected to test a less restrictive filter. The choice of filter band significantly affected classification performance, with the 1–20 Hz filter yielding the best results in the default model. The broader filter bands (0.2–20 Hz and 0.1–50 Hz) performed similarly and yielded the lowest MCC estimates overall.
(II) To examine impact in an online setting, the PRK trained model for each participant was tested in a simulation using data from the RSVP copy phrase task. The individual OF and CF PRK models performed similarly (see Figure 5), with a slight trend toward significance in the OF condition (*p* = 0.15).

## Discussion

In this study, we investigated the hypothesis that filter application with greater online parity would improve cBCI calibration performance. The results demonstrate that the OF was a suitable procedure for data pre-processing and resulted in small but significant improvements to model performance across model types. Furthermore, usage of models trained in this manner during online simulations demonstrated its viability.

To explain the differences in filtering results, several plots were presented alongside the statistical results. Alongside the default BciPy filter used, we presented data using several common filters advocated for in literature on the PRK model (Zhang et al., 2024a). As demonstrated in Figure 4, the ideal filter for this model appears to be 1-20 Hz, with the OF outperforming CF. In all other high- and low-pass conditions, performance dropped for both filtering approaches. By reducing the high pass filter, CF outperformed or matched OF results, however overall performance declined significantly. This suggests that the OF approaches produces a better filter for application of trials to be classified for usage online, particularly when tighter filters were used. Although exploring model-specific differences was outside of the scope of this manuscript, such an exploration would be a fruitful avenue for future research.

Reflecting on Zhang et al.’s (2024b) earlier filtering exploration, it is possible that both the N200 and P300 information are relevant for model classification; otherwise, a 1-10 Hz or lower high-pass filter might have produced the best results. While reducing high pass filtering to recommended settings did not increase classification performance, it may be the optimization for signal to noise ratio (SNR) other than amplitude measures (mean and peak) were more meaningful for classification. Furthermore, a model may be relying on frequency content or other derivations of the signal for its classification, while traditional ERP studies index amplitude to determine effects where high pass filtering could be destructive (Zhang et al., 2024a; Delorme, 2023). The difference in usage of underlying signal properties for evaluations highlights the need to explore standards of practice in one domain before transferring to another.

**Figure 6:**
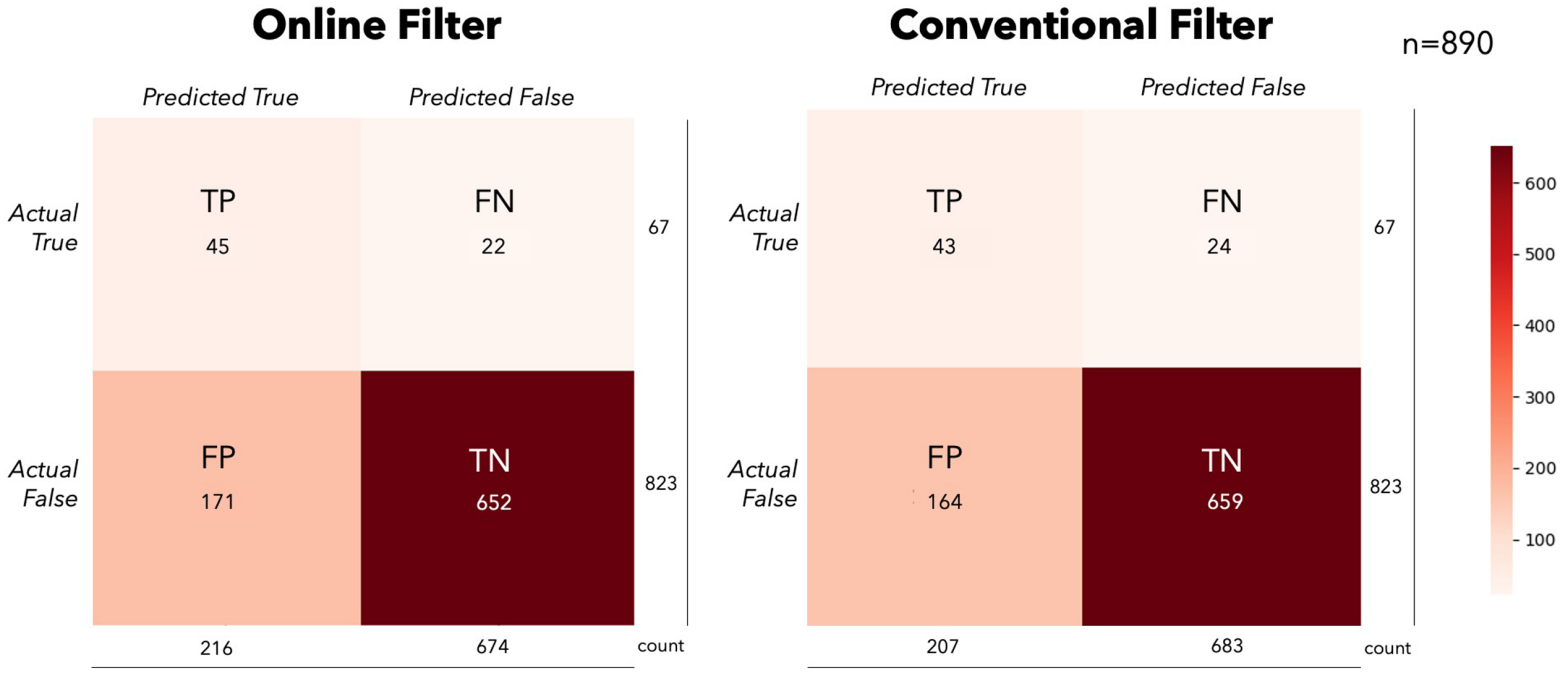
Online simulation confusion matrices. This figure presents the averaged confusion matrices for the online simulations in the OF (left) and CF (right) conditions. While there were no statistical differences in the primary performance measures (BA/MCC), slight differences in model performance can be seen on average. The OF model predicted more of the positive class on average, where CF favored the negative class.

The results of the simulation on performance between models suggests the different approaches are similar (see Figure 5). This comparability may not hold as filters become stronger (higher order) or tighter (smaller bands), as we observed in the different filtering bands during classification in the supplementary analyses. Furthermore, forcing likelihood measures into a confusion matrix may not be the ideal way to compare these conditions. The magnitude of the predictions may lead to faster typing performance, where decision thresholds and cumulative Bayesian updates would be more sensitive to these differences. However, this scenario would be difficult to simulate offline given the sequential nature of the task and lack of stationarity of the underlying signals. While outside the scope of this experiment, more could be done to estimate a typing rate change from real or simulated data using different modeling techniques. The confusion matrices show some slight differences in model performances on average, albeit insignificant in our simulations (See Figure 6). The OF model had more positive class identifications on average (TP), where CF did better on average at identifying the negative class (TN). The impact of false identification in either direction would depend heavily on the evidence fusion and gathering strategy. As already discussed above, for systems using Bayesian fusion, the certainty of that label would impact the system significantly, and therefore should be monitored closely when selecting models.

In this work, we performed analyses on a small sample of N200/P300-based BCI data. Our analysis may be informative and valuable for understanding an artifact-handling procedure’s potential benefits and limitations. However, it is essential to note that other types of EEG signals are used in BCIs, such as SSVEP or code-based visual evoked potentials, and sensory-motor rhythm. Future work may investigate how these findings generalize to other types of BCIs. In addition, the type of sensor and whether or not it is implanted should be considered when deciding the correct processing procedures. In this experiment, dry electrodes were used. Acknowledging this point, however, the N200/P300 BCIs are known to have lower SNR and are sensitive to filtering choices, making the analysis pragmatic to the field, albeit incomplete. Additionally, replicating these results with a greater number of participants and in different environments would be informative.

This study focused on one facet of the artifact pipeline, namely filter application. However, given these results and previous literature, more could be done to evaluate other strategies to mitigate artifacts online. While some studies have shown the process of artifact removal to be detrimental to performance measures (Thompson et al., 2019), it could be tested better online where some samples could be recollected to avoid data loss constraints. This was explained in some detail by Delorme (2023), where the removal of trials was beneficial only to a point. This was further demonstrated by Zhang et al. (2024a), where it was advantageous to remove artifacts; however in their dataset, removal never exceeded 5% of trials (Zhang et al., 2024b). Therefore, it may become crucial in the presence of select artifacts to collect more data instead of dropping trials altogether. Furthermore, to complement these investigations, more could be done to quantify the impact of different artifacts (blinks, electrode pops, etc.) and their removal on model prediction performance directly. An investigation does exist using simulated artifacts (Awais et al., 2024). This data could be used to help define the remedies for various types of noise encountered in cBCIs, as these signals could also be leveraged for increased performance in the case of properly timed eye activity (stimuli not missed by action) (Liu et al., 2024).

## Conclusion

This manuscript presented evidence that training a model in the same way it will be used online was a suitable approach and may be beneficial for long-term use. Supplementary analyses demonstrated the viability of this online parity pre-processing via simulation and provided evidence of the ideal bandpass filter for RSVP cBCIs in the 1-20 Hz range. Future studies should test this methodology online with varied artifact handling procedures, interfaces, and model types to determine transferability constraints of this approach across BCI systems.

## Data Availability

The raw data supporting the conclusions of this article will be made available by the authors, without undue reservation.
